# Spatiotemporal pattern and coordination relationship between urban residential land price and land use intensity in 31 provinces and cities in China

**DOI:** 10.1371/journal.pone.0254846

**Published:** 2021-07-20

**Authors:** Xingran Cai, Yanqing Liang, Zhiying Huang, Jingfeng Ge

**Affiliations:** 1 Northwest Institute of Eco-Environment and Resources, Chinese Academy of Sciences, Lanzhou, Gansu Province, China; 2 University of Chinese Academy of Sciences, Beijing, China; 3 College of Resources and Environmental Sciences, Hebei Normal University, Shijiazhuang, Hebei Province, China; 4 Hebei Technology Innovation Center for Remote Sensing Identification of Environmental Change, College of Resources and Environmental Sciences, Shijiazhuang, Hebei Province, China; 5 Hebei Key Laboratory of Environmental Change and Ecological Construction, College of Resources and Environmental Sciences, Shijiazhuang, Hebei Province, China; 6 School of Land Science and Space Planning, Hebei GEO University, Shijiazhuang, Hebei Province, China; Northeastern University (Shenyang China), CHINA

## Abstract

The trend towards efficient and intensive use of land resources is an inevitable outcome of current social development. The rational matching of urban land prices and land use intensity has become an important factor under accelerating urbanization, and promotes the healthy development of the social economy. Using data on residential land price and on land use intensity for 31 provinces and cities in China, we employ the E-G cointegration test and quadrant map classification to determine the coordination relationship between land price and land use intensity. We then employ HR coordination to calculate the coordination degree of land price and land use intensity, and classify the coordination type accordingly. Our results are as follows. (1) The spatio-temporal distribution of urban land price shows high variability with multiple maxima, and follows a decreasing trend from the southeast coastal area to the northwest inland area and the northeast. (2) The overall land use intensity is at or above the middle level, and shows large spatial differences between provinces, but the agglomeration between provinces is increasing. (3) From the perspective of the relationship between urban land price and land use intensity at the inter-provincial scale, we find that the land price and land use intensity are well coordinated, and the number of provinces has been dynamically changing during different development periods. There is an east-west difference in the spatial distribution of land price and land use intensity coordination level. Different provinces and cities with the same coordination stage show differences in their land price and land use intensity level.

## Introduction

As the space carrier of urban society, economy and environment, urban land use efficiency has an important impact on urban social development and the construction of human settlements [1–3]. Under accelerating urbanization and the increasing pressure of population growth, urban construction continues to expand, the land market is developing rapidly, and contradiction between increasing land demand and limited land resources has intensified [4–6]. Remaining problems in this respect include low efficiency of land use and wasted land resources, which hinder the development of the land market [7]. As an important metric reflecting the health of the land market, land price plays an increasingly important role in the national macro-control and resource optimization allotment [8]. Generally, for a higher level of urban land use intensity, the unit of available land area becomes smaller, the contradiction between land supply and demand increases, land price increases, and the land market has a greater impact [9]. As a result, China has successively promulgated a series of policy documents to strengthen the management and utilization of land resources. In 2008, “The notice of the state council on promoting the economical and intensive use of land” clearly proposed to strengthen the management of the utilization efficiency of commercial, residential and industrial land. In 2019, the Ministry of natural resources stressed the regulations on the economical and intensive use of land to ensure the decisive role of the market in the allocation of land resources and improve the strictest land-saving system. In addition, the revised “The land administration law of the people’s Republic of China” also promoted land market management to the level of national macro strategy. As a barometer reflecting the dynamic changes in the supply and demand of the land market, land price plays an important role in regulating land use type and scale, and in promoting land use intensity [10,11]. It is therefore of great theoretical and practical benefit to combine land price with land use intensity and to explore the evolution and coordination relationship between these two metrics; this can improve the stability of the land price market and promote efficient and intensive land use [12–14].

In the evaluation of urban land price and land use intensity, we consider four main characteristics, as follows. (1) Single-direction research. The spatiotemporal characteristics of land price or land use intensity are studies separately. The research on land price mainly focuses on spatial heterogeneity [15,16], spatiotemporal evolution [17–19] and influencing factors [20–24]. The study of land use intensity chiefly concentrates on the calculation of use intensity level [25–28], influencing factors [29,30] and the utilization measures [31]. (2) Interdisciplinary research. This explores the relationship between land price and land use intensity from qualitative and quantitative perspectives. Some researchers first separately analyzed the spatiotemporal differentiation characteristics of land price and land use intensity, and then assessed the relationship between land price and land use intensity in combination with the consistency of spatiotemporal characteristics [32]. (3) Single research scale. Here, the research scale is mostly at the small-scale level of counties and cities, with the aim of analyzing the relationship between land price and land use intensity, while the large scale, especially the national scale, remains to be further studied [33,34]. In this case, the methods usually include a linear regression model to determine the degree of correlation between the land price and land use intensity, as quantified by the regression coefficient [35]. (4) Few comprehensive research results. Previous studies have generally focused on the relationship between land price and land use intensity [36], but evaluating what kind of relationship exists between the two, and the degree of their relationship, still require further investigation. Consequently, we adopted a combination of qualitative and quantitative methods to study the relationship between land price and land use intensity, which not only explained the relationship between them from the qualitative point of view, but also verified the relationship between them from the quantitative point of view. According to previous existing research results, we considered the factors influencing urban land price and land use intensity, taking 31 provinces (cities) in China as a case to study, and employing the quadrant map classification and HR coordination model. This approach can reveal deep insights into the underlying drivers of land price and land use intensity, and provides a scientific basis with which the government can formulate relevant land policies and implement a differentiated urban construction land management scheme.

## Methods and materials

### Study area

**Fig 1:**
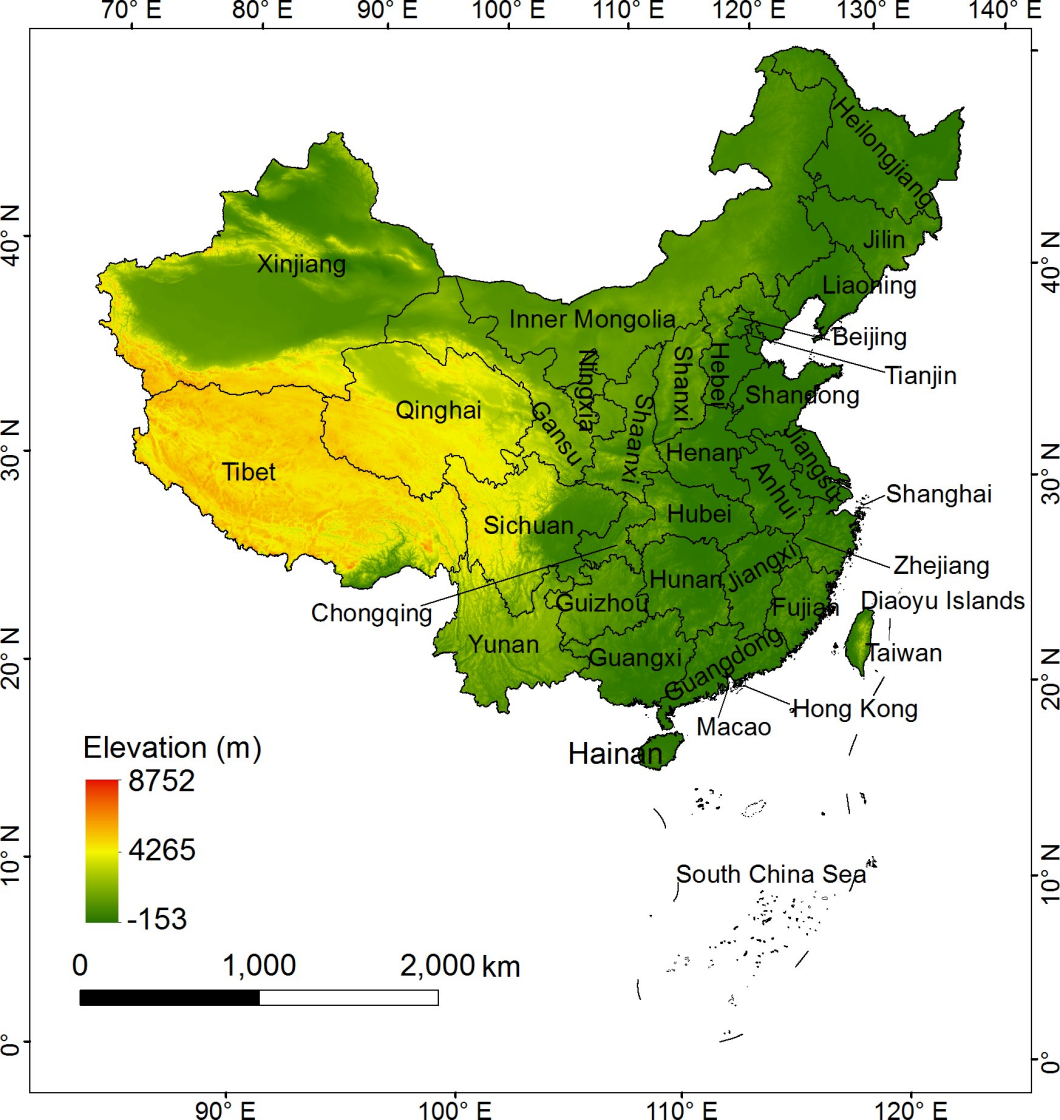
Location of the study area (the map was prepared in ArcGIS 10.1 using political boundaries from the National Geomatics Center of China (http://www.ngcc.cn/ngcc/) and Digital Elevation Model from the U.S. Geological Survey (https://www.usgs.gov/)).

China is located in the east of Asia and on the west coast of the Pacific Ocean at 3°52’~53°33’ N, 73°40’ ~135°2’ E. The terrain is high in the west and low in the east, with complex and diverse landforms, including plains, mountains, plateaus, basins and hills. The world’s highest plateau (the Qinghai-Tibet Plateau) and the world’s highest peak (Mount Everest) are both situated in China. The climate types are diverse, and the monsoon climate is significant. China has 34 provincial-level administrative regions, including 23 provinces, 5 autonomous regions, 4 municipalities directly under the central government and 2 special administrative regions. In 2019, China’s GDP reached 99086.5 billion yuan, an increase of 6.1% over the previous year (Fig 1).

### Methods

#### Digital land price model

A digital land price model is a surface model that expresses land prices formed by point coordinates. According to the characteristics of land price data, the Kriging interpolation method was selected to establish a digital land price model. The calculation formula is as follows:

$$Z(x_{0})=\sum_{i=1}^{N}y_{i}Z(x_{i})$$ (1)

where *Z*(*x*_*0*_) is the value of the unknown sample land price data, *N* is the number of the known sample land price data, *y*_*i*_ is the weight of the *i*th known sample land price data, and *Z(x*_*i*_*)* is the value of the known sample land price data.

#### Calculate land use intensity level

Evaluation of urban land use intensity is a complex system, involving many factors, so the establishment of the evaluation index system requires further consideration. We selected statistics from 30 relevant studies in the China Knowledge Resource Database that were related to the construction of the urban land use intensity index system from 2015 to 2017. Amongst the four dimensions of land use input, land use benefits, land use social benefits and land use sustainability, the most frequently cited indicators (frequency greater than 5) were selected to construct the evaluation index system [37–42]. To eliminate the influences of subjective factors, the entropy method was used to determine the index weight (Table 1).

**Table 1:** Evaluation index system of urban residential land use intensity.

Sub-index	Weight	Index	Weight
Land use input	0.34	Local total fixed assets investment	0.03
		Local real estate development investment	0.06
		Local technology expenditure	0.18
		Per capita urban road	0.06
Land use benefits	0.34	Local finance revenue	0.10
		Local the second and third industries	0.09
		Per capita gross domestic product	0.11
		Local retail sales of social consumer goods	0.04
Land use social benefits	0.21	Urban water utilization rate	0.01
		Urban gas penetration rate	0.01
		Local telephone traffic	0.10
		Local Number of hospital health institutions	0.09
Land use sustainability	0.10	Local industrial wastewater discharge	0.03
		Urban area green coverage rate	0.04
		Local sulfur dioxide emission	0.03

To determine the indicator weight, the multi-factor comprehensive weighted sum method was used as a measure of the level of land use intensity.

$$F(x)=\sum_{i=1}^{m}\left(W_{t}\times Y_{it}\right)$$ (2)

where *F(x)* is the level of urban land use intensity, *m* is the number of research objects, *W*_*t*_ is the weight of indictor *t*, and *Y*_*it*_ is the standardization of indictor *t*.

#### E-G cointegration test

The E-G cointegration test, which was proposed by Engle and Granger in 1987, is based on regression residuals [43]. Its main idea is that if land price can be explained by a linear function of land use intensity, there is a stable equilibrium relationship between them; in contrast, if the land price cannot be explained by land use intensity, then the residuals should form a stationary series.

- If there are *k* sequences, *y*_*1*_ and *y*_*2*_, *y*_*3*_, *y*_*4*_…, *y*_*K*_ is a single-order sequence, and the regression equation is established:
$$y_{1t}=\beta_{2}y_{2t}+\beta_{3}y_{3t}+\cdots +\beta_{k}y_{kt}+u_{t}\;t=1,2,\cdots ,T$$ (3)
- The residual of the regression equation is:
$${\hat{u}}_{t}=y_{1t}-{\hat{\beta }}_{2}y_{2t}-{\hat{\beta }}_{3}y_{3t}-\cdots -{\hat{\beta }}_{k}y_{kt}$$ (4)

*ADF* was used to test the stationarity of the residual series of the regression equation. If the residual of the regression equation for land price and land use intensity was shown to be a stationary sequence through the *ADF* test, we consider that there is a stable cointegration relationship between the *k* variables of the regression equation.

#### Quadrant map classification method

In terms of the relationship between urban land price and land use intensity, this study is based on the quadrant map classification method proposed by Chen et al [44]. Their approach is then combined with the degree of deviation method, which we use to explore the spatial link between urban land price and land use intensity. The process is summarized as follows.

1. Standardize the urban land price data (D) and land use intensity level data (F) again, to generate the new urban land price (ZD) and land use intensity level (ZF).
2. Taking the urban land price (ZD) as the abscissa and land use intensity level (ZF) as the ordinate, we constructed the quadrant map of the interaction between the urban land price and land use intensity. The urban land price and the land use intensity level in different years and different regions were assimilated into a point set (ZD, ZF), which was presented in the quadrant map in the form of scatter plot.
3. The relationship between urban land price and land use intensity was judged according to the location of scatter plot in the quadrant map. In the first quadrant, ZD > 0 and ZF > 0. Samples plotting in this quadrant indicate that urban land price and land use intensity level are both high, with a good coordination degree. In the second quadrant, ZF < 0 and ZD > 0. For these sample points, the urban land use intensity level is low and land price is high, and their development may reflect a low level of coordination or an imbalance. In the third quadrant, ZD < 0 and ZF < 0. Samples plotting in this quadrant indicate that urban land price and land use intensity level are low, with a low-level of coordinated development. In the fourth quadrant, ZF > 0 and ZD < 0. In this quadrant, urban land price is low and land use intensity level is high, and the development of both factors is at a poorer stage than those of the first and third quadrants.

#### HR coordination model

To discuss the degree of coordination between urban land price and land use intensity in more detail, we employed the HR coordination model proposed by Zhou and Kong [45]:

$$HR=1-\frac{S}{\bar{P}}$$ (5)

$$C_{v}=\frac{S}{\bar{P}}=\frac{\sqrt{\frac{{\left(P_{1}-\bar{P}\right)}^{2}+{\left(P_{2}-\bar{P}\right)}^{2}}{2}}}{\frac{\left(P_{1}+P_{2}\right)}{2}}$$ (6)

where *HR* is the degree of coordination between land price and land use intensity, *S* is the standard deviation of land price and land use intensity level, $\bar{P}$ is the arithmetic mean of land price and land use intensity level, and *C*_*v*_ is the coefficient of variation.

To ensure that *HR* is between 0 and 1, *C*_*v*_ must be between 0 and 1. However, since *C*_*v*_ does not necessarily satisfy this condition, we used the following adjustment:

$$HR=1-\frac{C_{v}}{C_{\max}}=1-\frac{1}{C_{\max}}\times \frac{\sqrt{\frac{{\left(P_{1}-\bar{P}\right)}^{2}+{\left(P_{2}-\bar{P}\right)}^{2}}{2}}}{\frac{\left(P_{1}+P_{2}\right)}{2}}$$ (7)

where *C*_*max*_ is the maximum value of *C*_*v*_, so $\frac{C_{v}}{C_{max}}$ is between 0 and 1. If *HR* = 1, land price and land use intensity are strongly coordinated; if 0 < *HR* < 1, land price and land use intensity are in a coordinated state; if *HR* = 0, land price and land use intensity are completely out of balance.

### Data acquisition

The land price data were obtained from the annual monitoring of residential land in 31 provinces in China, from 2007 to 2016, by the China urban land price dynamic monitoring network. Meanwhile, to reflect the spatial distribution pattern of urban land price more closely, 105 land price monitoring cities in China were selected for analysis. The indicator data of land use intensity came from the 2008–2017 China Statistical Yearbook, China City Statistical Yearbook and relevant statistical yearbooks of each province. Some missing data were calculated using the annual growth rate. The year 2007 was a period of rapid economic development in China, with obvious fluctuations of land price but a low degree of land use intensity. Under this background, China has established a national-level urban dynamic monitoring system to reflect the allocation of urban land resources and the development environment of the whole macro-market economy. From the perspective of land price changes from 2003 to 2007, the land prices of various land use types in major cities in China increased the fastest and changed the most obviously in 2007; in particular, the average increase of the residential land price was 9.17 percentage points higher than that in 2006. The year 2016 was the period when China’s Five-in-one layout was established, under which the land conservation and intensive utilization represented the fundamental strategy for the construction of ecological civilization. Meanwhile, China’s economic development has entered the “new normal”, with obvious land price growth, of which the residential land price has grown fastest. Based on this, we chose 2007 and 2016 as the start and end years respectively.

## Results

### Spatiotemporal characteristics of residential land price

**Fig 2:**
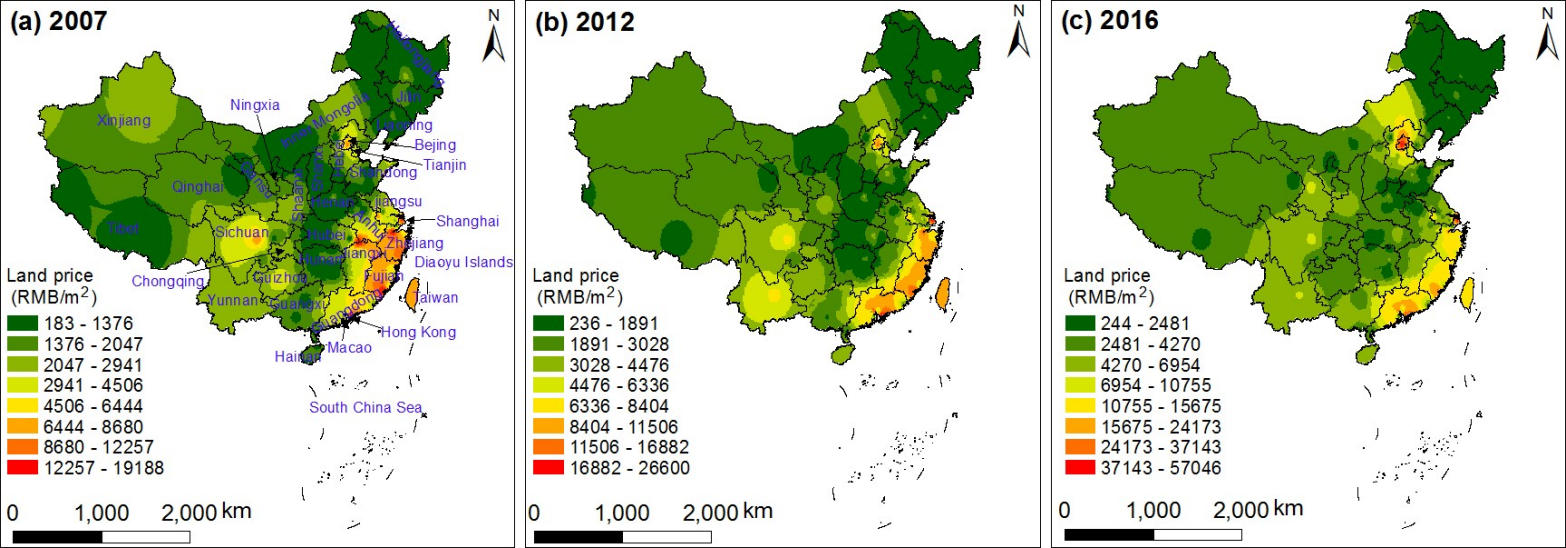
The provincial spatiotemporal pattern of urban residential land price (the map was prepared in ArcGIS 10.1 using political boundaries from the National Geomatics Center of China (http://www.ngcc.cn/ngcc/) and land price data from China urban land price dynamic monitoring network (http://www.landvalue.com.cn/)).

In recent years, the urban land price has shown a gradual upward trend. To reflect the trend of urban land price development under the level of social and economic development, and to maximize the use of available samples, 105 land price monitoring cities with data in 2007, 2012 and 2016 were used to analyze the spatial characteristics of residential land price (Fig 2).

The spatial distribution of urban land prices shows a distinct overall pattern, with a gradual decrease from the southeast coastal area towards the northwest inland area and the northeast area. The southeast coastal areas, especially the Pearl River Delta and the Yangtze River Delta, experienced accelerated local socio-economic development due to their advantageous geographical location and open policies. The rapid economic development led to an increase in the local population, and increasingly scarce land resources, which in turn drove the increase in the residential land price. The northwest region is located in the mainland of China. Limited by its environmental conditions, the large land area is sparsely populated, and the transportation facilities such as roads and railways are poor. Under the influences of these natural and social conditions, the land price in this area was low. Similarly, because of the high latitude and low temperature in winter in Northeast China, the population tended to move to other areas. The migration of population and the slow development of the social economy led to the low land price.

Here we discuss the coexistence and distribution of "multiple high-value" centers of urban land price. Beijing and Tianjin were high price centers in North China, from which land prices decreased outwards with increasing distance. As the capital of China, Beijing is the national center of political, economic, social, technological and cultural development. With its dense population, extensive infrastructure and relatively high level of economic development, land price has risen rapidly. Tianjin is close to Beijing and is located on the Bohai Sea coast. It is a famous port city with convenient land and sea transportation. Its unique port economy has attracted a large number of people, leading to a greater population density and increase in local land prices. Hebei and Shanxi have been driven by economic growth in Beijing and Tianjin, and their land prices have risen accordingly. In particular, Langfang and Tangshan in Hebei were more strongly affected by Beijing and Tianjin; as a result, Hebei has been more affected by Beijing and Tianjin than Shanxi has been. Overall, the land price gradually decreases in the order Beijing-Tianjin-Hebei-Shanxi. Shanghai is the center of an extreme land price peak in East China, owing to its well-developed economy and concentrated urban distribution. This is especially the case in Hangzhou of Zhejiang, and in Nanjing of Jiangsu, which are closely linked with Shanghai and have strong economic interdependence. The east of Anhui lies close to Jiangsu and Zhejiang, which has led to the development of cities, and the land price has increased slightly. Therefore, land price in East China showed a gradually decreasing trend away from Shanghai, in directions towards the north and south sides of Jiangsu and Zhejiang, and eastwards towards Jiangsu and Anhui. Guangdong has a superior geographical location, close to Hong Kong, Macao and Taiwan, with well-developed transportation, preferential policies and a high level of science and technology. Under the influence of these factors, it readily attracted social and economic investment and talents, and became the high-value center of land prices in South China. Fujian and Guangxi are adjacent to Guangzhou. Through their active involvement in industry expanding outwards from Guangzhou, their economic ties became close, promoting an increase of land prices in Fujian and Guangxi. Therefore, land price in South China showed a decreasing trend towards Guangzhou, and an increase towards Fujian and Guangxi on the east and west sides.

### Spatiotemporal characteristics of land use intensity

**Fig 3:**
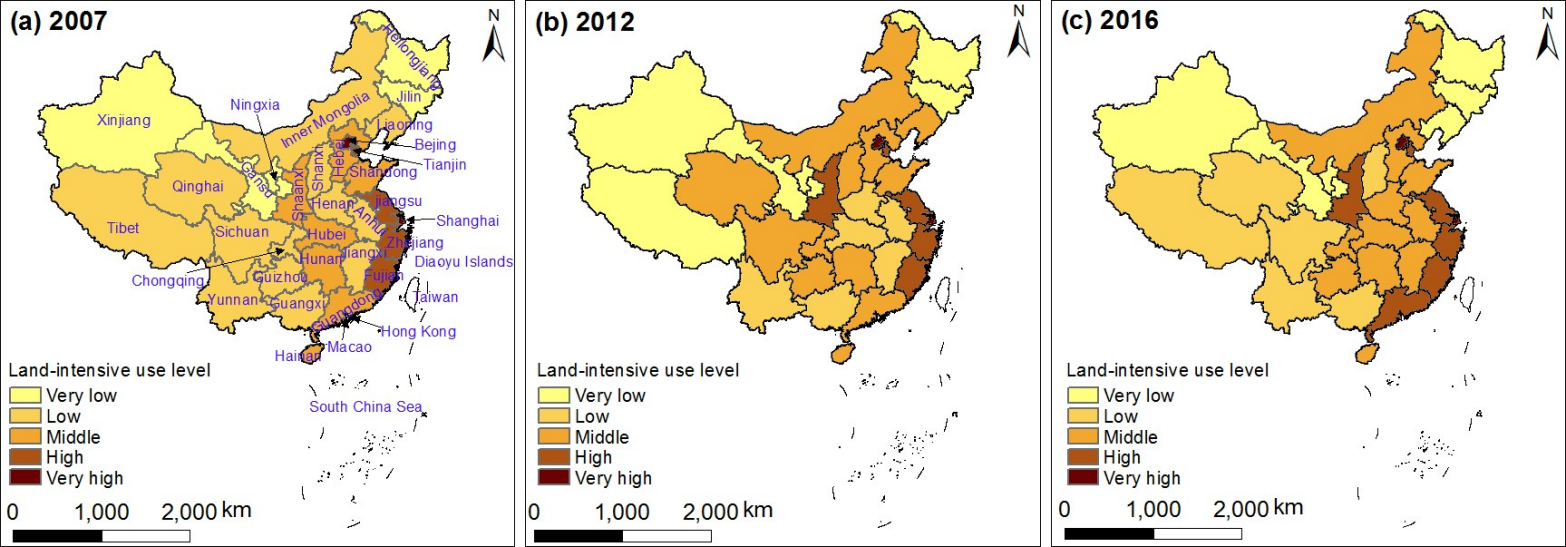
The provincial spatiotemporal pattern of urban residential land use intensity (the map was prepared in ArcGIS 10.1 using political boundaries from the National Geomatics Center of China (http://www.ngcc.cn/ngcc/) and land use intensity data from relevant statistical yearbook).

With the significant increase in the level of urbanization, the level of urban land use intensity has also changed spatially. Based on the natural breakpoint method implemented in Arcgis 10.1 software, the urban land use intensities of 31 provinces in China in 2007, 2012 and 2016 were divided into five levels (very low, low, middle, high and very high), so as to obtain the provincial spatial-temporal pattern of urban land use intensity level (Fig 3):

The predominant overall land use intensity level was in the middle or higher levels. According to the comparative analysis of land use intensity levels in 2007, 2012 and 2016, the number of provinces with low or below level in 2007 decreased from 18 to 12 in 2012 and then to 11 in 2016, while the number of provinces with middle and above level increased from 13 to 19 and then to 20, indicating that urban land use intensity in China has developed from the very low and low levels to the middle and higher levels. However, the numbers of high level and very high level provinces were small. Therefore, when considering future land use, it is necessary to further improve land resource utilization efficiency to realize the efficient, economical and intensive use of residential land.

Significant differences were observed in the spatial distribution of land use intensity level. The high and very high levels of land use intensity were mainly distributed in the southeast coastal areas; Beijing and Shanghai have maintained particularly high land use efficiency, followed by Guangdong, Zhejiang, Jiangsu, and Fujian. Cities with the middle level of land use intensity were mainly concentrated in the central region. Affected by Beijing and Tianjin, Yangtze River Delta and Pearl River Delta, we deduced that Hebei, Shandong and other places experienced significantly increased input levels, output benefit and utilization intensity in the process of land resource use, leading to an evident effect on land use intensity. Cities with low and very low levels of land use intensity were mainly distributed in the western and northeastern regions. Here, the western region has rugged terrain and less available land. In addition, due to the low local economic level, few people were encouraged to relocate to this region, the input level of land resource utilization was low, and the utilization rate of land resource was generally low. As a famous development base of heavy industry in China, the northeast region has gradually lost its development advantage, leading to under-utilized land resources and low allocation efficiency with the acceleration of national industrial structure adjustment in recent years. In this context, and compared with other regions, the land use intensity level in Northeast China was obviously lower.

The spatial agglomeration of land use intensity level has increased. In 2007, the spatial distribution of land use intensity in China presented a four-level distribution, which showed that Jiangsu-Zhejiang-Fujian was at the highest level, while Inner Mongolia-Shanxi-Henan-Anhui-Jiangxi was the lowest level area. Shanxi-Hubei-Hunan-Guangdong-Hainan was at the middle level, and Qinghai-Sichuan-Yunnan was at a very low level. The low level area surrounded the middle level area. In 2012, the spatial distribution of urban residential land use intensity level was relatively scattered, showing a predominantly two-level distribution, with Shanxi-Hebei-Shandong at the middle level, and Sichuan-Chongqing-Hubei-Anhui at the low level. In 2016, the spatial distribution of land use intensity level presented the above-mentioned “four vertical and two horizontals” crisscross pattern.

### Coordination between urban residential land price and land use intensity

Based on the analysis of the characteristics of urban residential land price and land use intensity, this paper used the E-G cointegration test, quadrant map classification method and HR coordination model for quantitative analysis to study the spatial relationship between them and the evolution characteristics of inter-provincial spatial pattern.

In order to verify the relationship between urban residential land price and land use intensity, based on ADF test and E-G cointegration test, Eviews 8.0 software was used to test the cointegration between urban residential land price and land use intensity. Result indicated that the ADF of residential land price and land use intensity was -2.01, less than -1.99 and P was 0.05 at the 5% confidence level, which formed a stable sequence, demonstrating that there was a stable cointegration relationship between residential land price and land use intensity.

#### Coordinated relationship between urban residential land price and land use intensity

**Fig 4:**
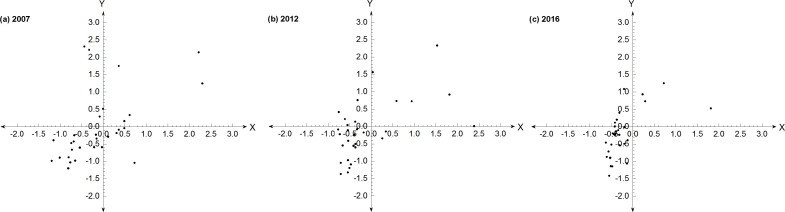
Quadrant map of the coordination relationship between urban residential land price and land use intensity (the map was prepared in Origin using reanalysis data of land price and land use intensity).

Fig 4 indicates that there were more provinces in the first and third quadrants, suggesting that the relationship between urban residential land price and land use intensity was well coordinated in most provinces. The provinces in the first or third quadrant were mainly distributed in eastern coastal areas, as well as in central and western parts of China, such as Beijing, Shanghai, and Guangdong, showing that these regions had achieved a relatively high level of coordinated development in residential land price and land use intensity. The provinces represented by Hubei, Hunan, and Sichuan showed a low level of coordinated development. The provinces in the second or fourth quadrants had land prices that led or lagged the land use intensity changes; these provinces were mainly located in the western and central parts of China, and included Ningxia, Qinghai, and Fujian, where land price changes led land use intensity. In the process of urban land price growth, we should constantly improve the land use structure and spatial pattern, and strive to increase the benefits gained from the limited land resources, so as to maximize the potential land use intensity. Under the guidance of this goal, the provinces in the second and fourth quadrants of the urban residential land price and land use intensity in China were consistently transformed to the first or third quadrant, revealing that the extent of the coordinated development of urban land price and land use intensity gradually expanded to the western and northeast regions. Although some provinces in the western or northeast regions were still at a low level of coordination, the coordinated development trend was increasing.

#### Pattern of coordination between urban residential land price and land use intensity

The quadrant map classification method used to study the coordination relationship between urban residential land price and land use intensity can clarify whether the urban residential land price and land use intensity were coordinated, but the degree of coordination and imbalance between them cannot be judged. Therefore, the coordination degree was calculated using the HR coordination model. In light of relevant research and when considering the state of development of the country, the degree of coordination was classified as one of five categories: severe imbalance, weak imbalance, basic coordination, good coordination and extreme coordination, as shown in Table 2.

**Table 2:** Coordination type of urban residential land price and land use intensity.

Coordination type	Coordination degree	Comparison of indicator types		
		*D*-*F*>0.1	│*D*-*F*<0.1│	*F*-*D*>0.1
Severe imbalance	0≤HR<0.2	Lag of land intensive use	Synchronous	Lag of land price
Weak imbalance	0.2≤HR<0.4	Lag of land intensive use	Synchronous	Lag of land price
Basic coordination	0.4≤HR<0.6	Lag of land intensive use	Synchronous	Lag of land price
Good coordination	0.6≤HR<0.8	Lag of land intensive use	Synchronous	Lag of land price
Extreme coordination	0.8≤HR<1	Lag of land intensive use	Synchronous	Lag of land price

Note: The comparison of indicator types includes a lag of land use intensity (land price is higher than land use intensity index), synchronous change (the difference between the absolute values of land price and land use intensity index is within 0.1) and lag of land price (land price is less than land use intensity index). Based on this classification, we conducted a comparative analysis of land price and land use intensity again to clarify whether the coordination types lead, lag or are synchronous with the land use intensity.

**Fig 5:**
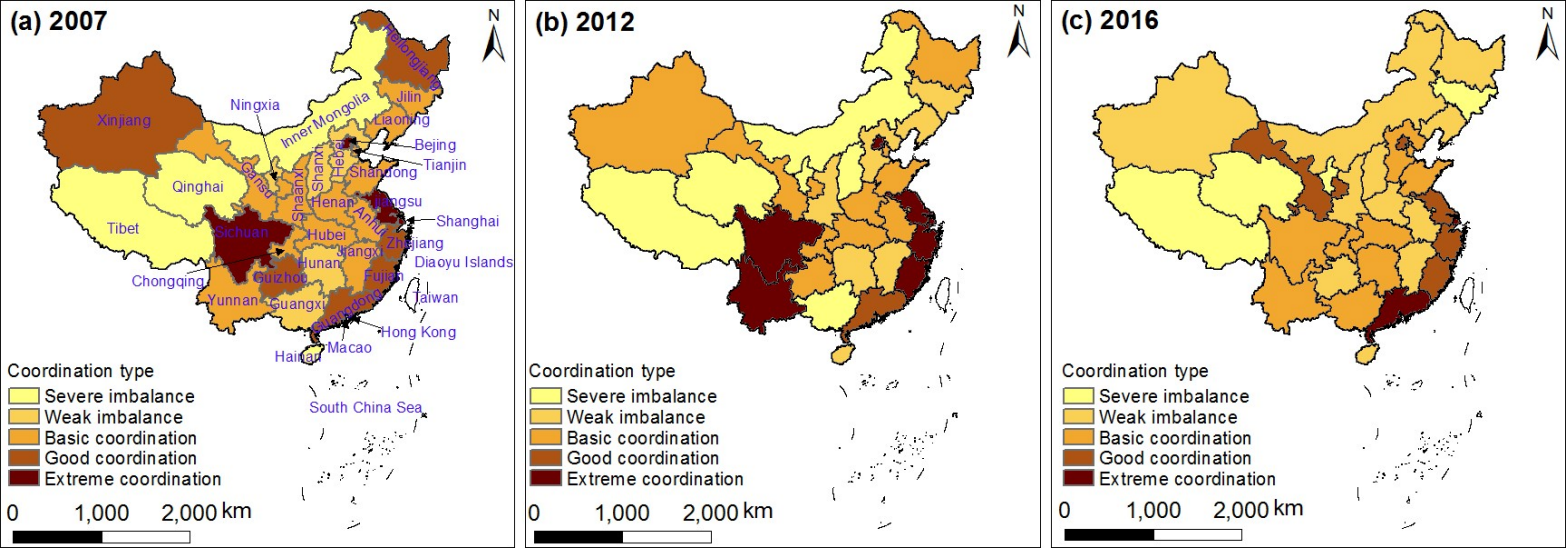
The spatiotemporal pattern of coordination type of urban residential land price and land use intensity (the map was prepared in ArcGIS 10.1 using political boundaries from the National Geomatics Center of China (http://www.ngcc.cn/ngcc/).

Combined with the standard of coordination degree between urban residential land price and land use intensity, the provincial spatial-temporal patterns of coordination degree in 2007, 2012 and 2016 were visualized using Arcgis 10.1 software (Fig 5).

Results revealed significant differences in coordination degree between land price and land use intensity in different development periods. The coordinated development of urban land price and land use intensity was a dynamic evolution process. Comparing the degree of coordination between residential land price and land use intensity in 2007, 2012 and 2016, we can see that the numbers of provinces exceeding the basic coordination stage were 23, 16, and 25, respectively. Despite the decrease from 2007 to 2012, most provinces were above the basic coordination stage in the past decade. This is because land prices in China were at a low level in 2007. As industry was adapting to economic development at that time, land resources were used reasonably, and the two factors showed a low level of coordinated development. Under the background of China’s rapid economic development in 2012, the land price increased rapidly and populations tended to become concentrated in big cities. Influenced by increasing land demand, the city scale was constantly expanding, the availability of land resources was insufficient, and the full utilization was not realized. Consequently, the increase in land price led the increase in land use intensity, which affected their mutually coordinated development. In 2016, the land price continued to increase, people’s awareness of land use intensity was enhanced, and the government continued to issue relevant policies on land use planning and protection measures, which increased the intensity of land investment. Therefore, the degree of land use intensity was higher, and even became excessive: this had a negative impact on social, economic and industrial progress. To some extent, the changes in land use intensity were ahead of land price, and this affected their coordinated development.

The spatial distribution of coordination degree between land price and land use intensity was different. Taking 2016 as an example, the provinces at the good or extreme coordination stages were mainly located in the eastern coastal area; the provinces at the basic coordination stage were mainly distributed in the southwest; and the provinces at the severe or weak imbalance stage were mainly scattered in the western inland region, the northeast, and some other eastern regions. Hence the spatial pattern of coordination degree of land price and land use intensity was characterized by decreasing coordination from the southeast coastal area to the northwest inland area.

Different regions at the same coordination stage showed obvious differences in terms of their coordinated development. There were divergences in land price and land use intensity in different regions, even when at the same coordination stage. For instance, Gansu and Jiangsu were in the extreme coordinated stage, but the degree of coordination was obviously different, for the following main reasons. Jiangsu’s land price and land use intensity were relatively high (in 2016, land price and land use intensity were 0.34 and 0.44 respectively), and classified as extremely high coordination; Gansu’s land price and land use intensity were relatively low (in 2016, land price and land use intensity were 0.28 and 0.20 respectively), with extremely low coordination. Jiangsu is an innovative development region in southern China, with a large number of technological companies, talented workforce, many capital resources, and advanced service industry and manufacturing; thus, while the land price rises, the land resources are reasonably planned and effectively utilized, and the two are in a highly coordinated stage. In contrast, Gansu is located in the inland region of western China, where the natural environment and workforce are suboptimal, the level of manufacturing industry is relatively low, and the output benefit is low. While the land price is slowly increasing, the land resource utilization efficiency is low, and the two are poorly coordinated.

## Discussion

### Validity of land price data and evaluation method

There are 105 land price monitoring cities in China. The number of these cities varies between the 31 provinces, from one to typically 9. To reflect the land price of each province most objectively, the land price data for the 27 provinces and 4 municipalities are represented by national first-level administrative regions, namely provincial capital cities and municipalities, according to China’s administrative divisions. Although they are representative, they are inevitably subject to certain restrictions.

In the construction of the land use intensity evaluation index system, the frequency analysis method is used to select the indicators, which has a certain degree of objectivity. Considering the integrity and differences of the provinces in China, additional scientific methods should be adopted in future research to build the index system, so as to reflect the land use status of residential land more comprehensively.

Quantitative analysis of residential land prices and land use intensity is achieved using the E-G cointegration test and quadrant map classification method. The HR coordination model can objectively reflect the status and trend of coordinated development between the land price and land use intensity, but this coordination needs exploring more deeply. Whether other methods or models can be used for spatial relationship analysis in the future is worthy of further discussion.

### Limitation and outlook

The relationship between land price and land use intensity has been a long-term focus of research. Here, we used exploratory data analysis, the E-G cointegration test, and related quantitative methods, such as the study of the relationship and its spatial and temporal characteristics, to gain a preliminary understanding of the interaction relationship between land price and land use intensity. This relationship was analyzed to some extent, but needs to be evaluated in more detail. In addition, there are regional differences in the coordination relationship between land price and land use intensity, which will be the focus of the next research. This ongoing work will quantify the effects of various influencing factors on the coordination relationship between land price and land use intensity and will help to realize coordinated and balanced development in the different regions.

## Conclusion

With the support of exploratory data analysis and index system construction, this paper analyzed the characteristics of residential land price and land use intensity in China’s inter-provincial cities. Through the E-G cointegration test, quadrant map classification method and HR coordination model, we discussed the coordination relationship, spatial distribution of coordination, and the evolution of coordination degree of residential land price and land use intensity in China.

Our results suggested wide spatial variability in land price, with a gradual decrease from the southeast coastal area to the northwest inland region and the northeast. Centers of high land prices were noted in Beijing, Shanghai and Guangzhou, representing the extreme land value areas in North China, East China and South China respectively. Overall, land use intensity was mostly at the middle level or above, with significant spatial differences. The high and very high levels were mainly distributed in the southeast coastal area, while the low and very low levels were mainly located in the western and northeast regions. However, the spatial agglomeration was becoming enhanced and presented a crisscross development trend.

In terms of the relationship between land price and land use intensity, the provinces in the first or third quadrants of land price and land use intensity were mainly distributed in the eastern coastal, central and western parts of China, while the provinces in the second or fourth quadrants were mainly located in the western and central regions of China. Due to the different coordination degrees between land price and land use intensity, there were apparent spatiotemporal differences amongst these regions. Over time, the number of provinces above the basic coordination stage fluctuated. The level of coordination decreased from the southeast coastal area to the northwest inland area. In addition, different regions at the same coordination stage presented clear divergence in terms of their coordinated development, which was manifested as high-level extreme coordination or low-level extreme coordination.

## Supporting information

S1 Fig Land price raster data of China in 2007, 2012 and 2016. The figure shows the spatial distribution of land prices across China in 2007, 2012 and 2016.
(XLS)

S2 Fig Land use intensity data of China in 2007, 2012 and 2016. The figure shows the spatial distribution of land use intensity across China in 2007, 2012 and 2016.
(XLS)

S3 Fig Land price and land use intensity data of China in 2007, 2012 and 2016. The figure shows the coordination relationship between land price and land use intensity across China in 2007, 2012 and 2016.
(XLS)

S4 Fig Land price and land use intensity data of China in 2007, 2012 and 2016. The figure shows the coordination type of land price and land use intensity across China in 2007, 2012 and 2016.
(XLS)

S1 Table Data of land use intensity in China. This includes the original data of 15 land use intensity evaluation indicators of China’s 31 provincial capital cities in 2007, 2012 and 2016.
(XLS)
